# Characterization of antimicrobial resistance and virulence traits of clinical *Pseudomonas mosselii* isolates with reduced meropenem susceptibility and preserved imipenem susceptibility

**DOI:** 10.1128/spectrum.03560-25

**Published:** 2026-05-28

**Authors:** Kaiqi Zeng, Lan Ma, Rong Zhang, Hongwei Gao, Jiachang Cai, Yanyan Hu

**Affiliations:** 1 Clinical Microbiology Laboratory, The Second Affiliated Hospital of Zhejiang University, School of Medicine, Zhejiang University 12377, Hangzhou, China; 2 Laboratory Medicine Center, The Second Hospital & Clinical Medical School, Lanzhou University 74713, Lanzhou, China; Second Affiliated Hospital of Soochow University, Suzhou, China

**Keywords:** *Pseudomonas mosselii*, reduced meropenem susceptibility, efflux pump, *oprM*, *tolC*

## Abstract

*Pseudomonas mosselii* is an environmental bacterium with increasing reports of pathogenic potential. Although carbapenems are considered highly effective against this species, emerging resistance presents a potential therapeutic concern. In this study, *P. mosselii* isolates exhibiting reduced susceptibility to meropenem (MEM) while remaining susceptible to imipenem (IPM) were identified from fecal samples of patients collected in 2023 at a tertiary hospital in Lanzhou, Gansu Province, China. Antimicrobial susceptibility testing confirmed the discrepant carbapenem susceptibility profiles. Whole-genome sequencing and comparative genomic analyses were performed to elucidate the underlying mechanisms. Among 63 isolates, 15 exhibited reduced susceptibility to MEM and were selected for further analysis. Genomic analysis revealed that the classical carbapenem-resistance determinant *oprD* remained intact, whereas mutations were observed in efflux pump-associated genes (*oprM*, *tolC*, and *ttgC*). In addition, virulence gene profiling demonstrated the conserved presence of determinants associated with alginate biosynthesis (*algD*, *algU*, *mucD*), motility (*fleQ*, *fliA*), type IV pili (*pilH*, *pilJ*), type VI secretion system (*hcp1*, *vipA/vipB*, *clpV1*), and siderophore production (*pvdH*, *pvdM*, *pvdS*), indicating a shared virulence potential among the isolates. These findings suggest that efflux pump alterations rather than *oprD* inactivation may contribute to the unusual phenotype characterized by reduced meropenem susceptibility and preserved imipenem susceptibility in *P. mosselii*. This study provides the first genomic insight into this unique carbapenem susceptibility pattern and broadens understanding of carbapenem resistance diversity in Pseudomonas species.

**IMPORTANCE:**

This study reports an unusual carbapenem susceptibility pattern in *P. mosselii* clinical isolates, characterized by reduced susceptibility to meropenem while remaining susceptible to imipenem and explores its potential underlying mechanisms. The findings reveal that efflux pump variations, rather than defects in *oprD*, may drive this unusual phenotype. As *P. mosselii* is typically susceptible to carbapenems, the emergence of isolates with reduced meropenem susceptibility highlights an overlooked antimicrobial resistance risk. Understanding this atypical mechanism provides valuable insights into the evolutionary plasticity of Pseudomonas and may guide future surveillance and treatment strategies against carbapenem resistance.

## INTRODUCTION

*Pseudomonas mosselii*, a member of the *Pseudomonas putida* complex, was first isolated from clinical samples in 2002 when it was described and named (1). However, it is a Gram-negative, non-fermentative bacterium widely distributed in the environment, particularly in soil and paddy fields (2). Notably, because of its ability to produce insecticidal proteins, this species has also been widely applied in the biological control of agricultural pests (2–4). Although it is infrequently isolated as a clinical pathogen, its pathogenic potential has been increasingly recognized, with reports indicating that it can cause sepsis and may also lead to invasive infections such as endocarditis in immunocompromised patients (5, 6). Moreover, *P. mosselii* has been reported to carry resistance genes including *bla*_VIM-1_ and *bla*_VIM-2_ (7, 8), indicating its potential relevance in nosocomial infections and the dissemination of antimicrobial resistance.

Carbapenems represent the last line of defense against severe Gram-negative infections, particularly those caused by extended-spectrum β-lactamase (ESBL)-producing Enterobacterale, owing to their stability against β-lactamases and broad-spectrum activity (9). Among them, meropenem (MEM) and imipenem (IPM) are the most widely used representatives (10). However, resistance to carbapenems has been rising globally, posing a major public health challenge (11). The underlying mechanisms are multifactorial, including carbapenemase production, porin loss, or modification, efflux pump overexpression, and alterations in penicillin-binding proteins (12). Importantly, phenotypic differences in MEM and IPM susceptibility may occur due to distinct molecular properties and permeability: OprD porin deficiency more strongly impairs IPM activity, whereas upregulation of efflux pumps such as MexAB-OprM more often contributes to MEM resistance (12).

Although carbapenem resistance has been extensively studied in major pathogens such as *Pseudomonas aeruginosa* and *Acinetobacter baumannii* (13), it remains poorly characterized in many less-common species, including *P. mosselii*. In 2023, we analyzed fecal samples from hospitalized patients in Gansu Province, China, and found extensive colonization by *P. mosselii*. Notably, a proportion of the isolates exhibited an atypical carbapenem susceptibility pattern, characterized by reduced susceptibility to MEM while retaining susceptibility to IPM. To date, limited knowledge is available regarding carbapenem resistance in *P. mosselii*. In particular, the occurrence of differential resistance phenotypes and the molecular mechanisms underlying them remain poorly understood. Here, we systematically investigated the antimicrobial susceptibility characteristics of selected *P. mosselii*, with particular emphasis on the prevalence and determinants of the atypical phenotype, focusing on carbapenemases, porins, and efflux pumps. These findings provide important insights into the resistance traits of *P. mosselii* and have implications for clinical therapy and infection control.

## RESULTS

### Bacterial distribution and antimicrobial susceptibility profiles

A total of 190 fecal samples were collected from hospitalized patients in Gansu Province, China, among which 63 *P. mosselii* isolates were identified (33.2%). Fifteen isolates showing reduced susceptibility to meropenem in the initial screening were selected for further investigation and subsequently confirmed by minimum inhibitory concentration (MIC) testing. Among these isolates, strain GS125 was isolated from the fecal sample of a 2-month-old infant, while strains GS151 and GS169 were recovered from newborns immediately after birth. The remaining isolates were obtained from adult patients.

The selected isolates displayed highly consistent antimicrobial susceptibility profiles based on MIC testing according to Clinical and Laboratory Standards Institute (CLSI) M100-S33 breakpoints for other non-Enterobacterales. All isolates exhibited high-level resistance to aztreonam, with all isolates being non-susceptible to piperacillin, whereas non-susceptibility to levofloxacin, cefepime, and ciprofloxacin was observed in 66.7%, 66.7%, and 26.7% of the isolates, respectively. In contrast, these isolates remained susceptible to ceftazidime, amikacin, and tobramycin. The detailed antimicrobial susceptibility results are presented in Table 1.

**TABLE 1:** Antimicrobial susceptibility of 15 MEM-R *P. mosselii* strains^*a*^

Strains	MIC (mg/L)												
	MEM	IPM	PIP	CAZ	SCF	FEP	ATM	AMK	TOB	CIP	LEV	DOX	MIN
GS5	8	1	32	16	≥64	≥32	≥64	8	≤1	2	4	8	≥16
GS10	32	1	64	16	≥64	≥32	≥64	≤2	≤1	1	4	8	8
GS11	8	0.5	32	16	≥64	16	≥64	≤2	≤1	0.5	4	4	4
GS19	>32	1	32	8	≥64	8	≥64	≤2	≤1	≤0.25	1	2	4
GS30	>32	2	32	16	≥64	≥32	≥64	8	≤1	2	4	8	8
GS39	8	1	32	8	≥64	4	≥64	≤2	≤1	≤0.25	1	2	2
GS44	16	1	32	16	≥64	≥32	≥64	≤2	≤1	1	4	4	8
GS67	>32	2	64	16	≥64	≥32	≥64	≤2	≤1	1	4	4	8
GS91	16	2	32	16	≥64	≥32	≥64	8	≤1	2	≥8	8	≥16
GS98	32	1	≥128	16	≥64	≥32	≥64	≤2	≤1	1	4	8	8
GS125	>32	2	64	16	≥64	≥32	≥64	≤2	≤1	1	4	8	8
GS149	>32	1	64	16	≥64	8	≥64	≤2	≤1	≤0.25	1	2	4
GS151	8	0.5	32	2	≥64	8	≥64	≤2	≤1	≤0.25	1	1	≤1
GS166	16	2	32	16	≥64	≥32	≥64	8	≤1	2	≥8	8	8
GS169	16	1	32	16	≥64	8	≥64	≤2	≤1	≤0.25	1	2	4

^*a*^ MEM, meropenem; IPM, imipenem; PIP, piperacillin; CAZ, ceftazidime; SCF, cefoperazone; FEP, cefepime; ATM, aztreonam; AMK, amikacin; TOB, tobramycin; CIP, ciprofloxacin; LEV, levofloxacin; DOX, doxycycline; MIN, minocycline. MICs of MEM and IPM were determined by the Etest method, while MICs of all other antimicrobial agents were determined using the VITEK 2 Compact automated system (bioMérieux, France).

### Molecular characteristics of selected *P. mosselii* isolates

**Fig 1:**
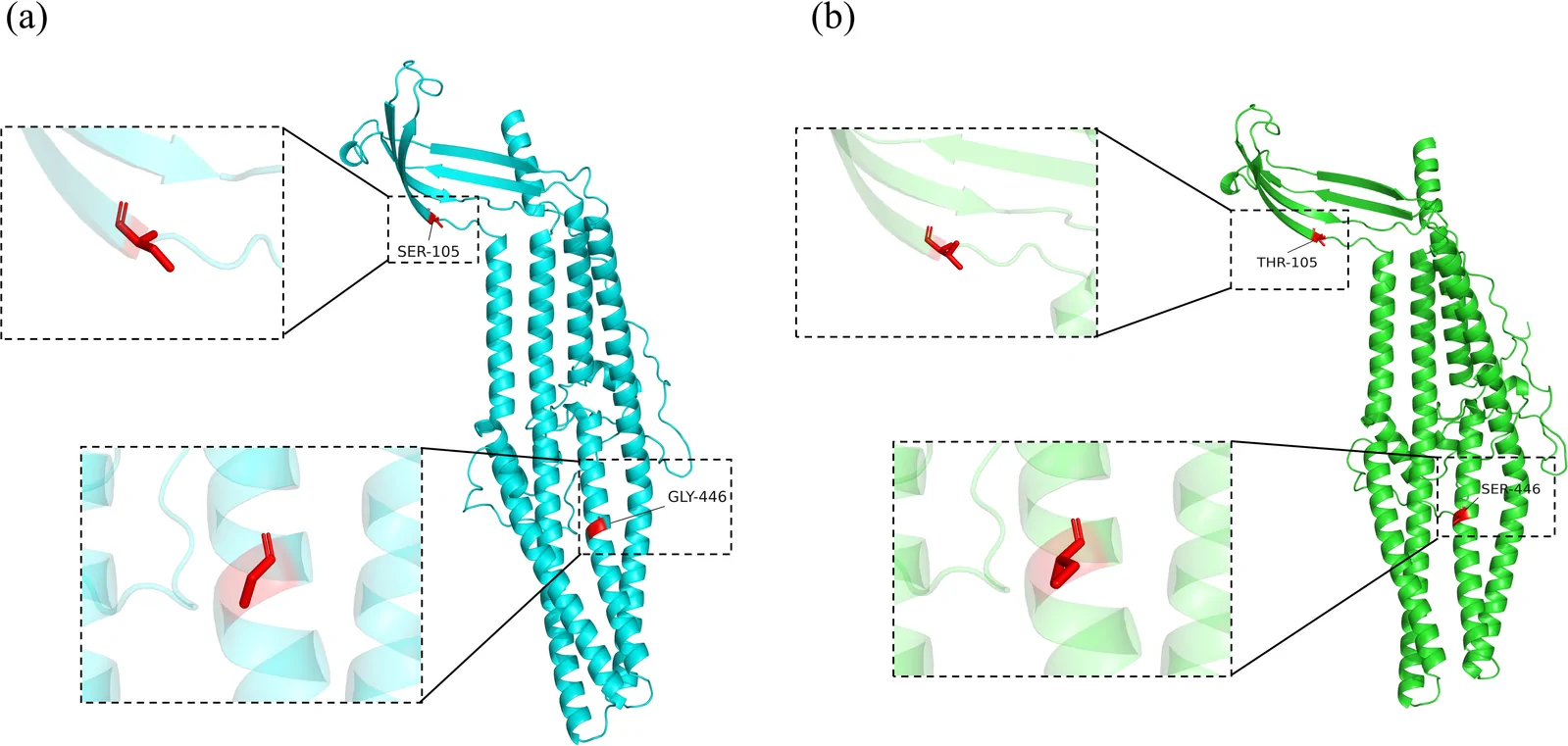
Comparison of three-dimensional structural models of wild-type (**a**) and mutant OprM proteins (**b**). The target residues at positions 105 and 446 are indicated in red. The enlarged insets show the local structural environments of the substituted residues.

**Fig 2:**
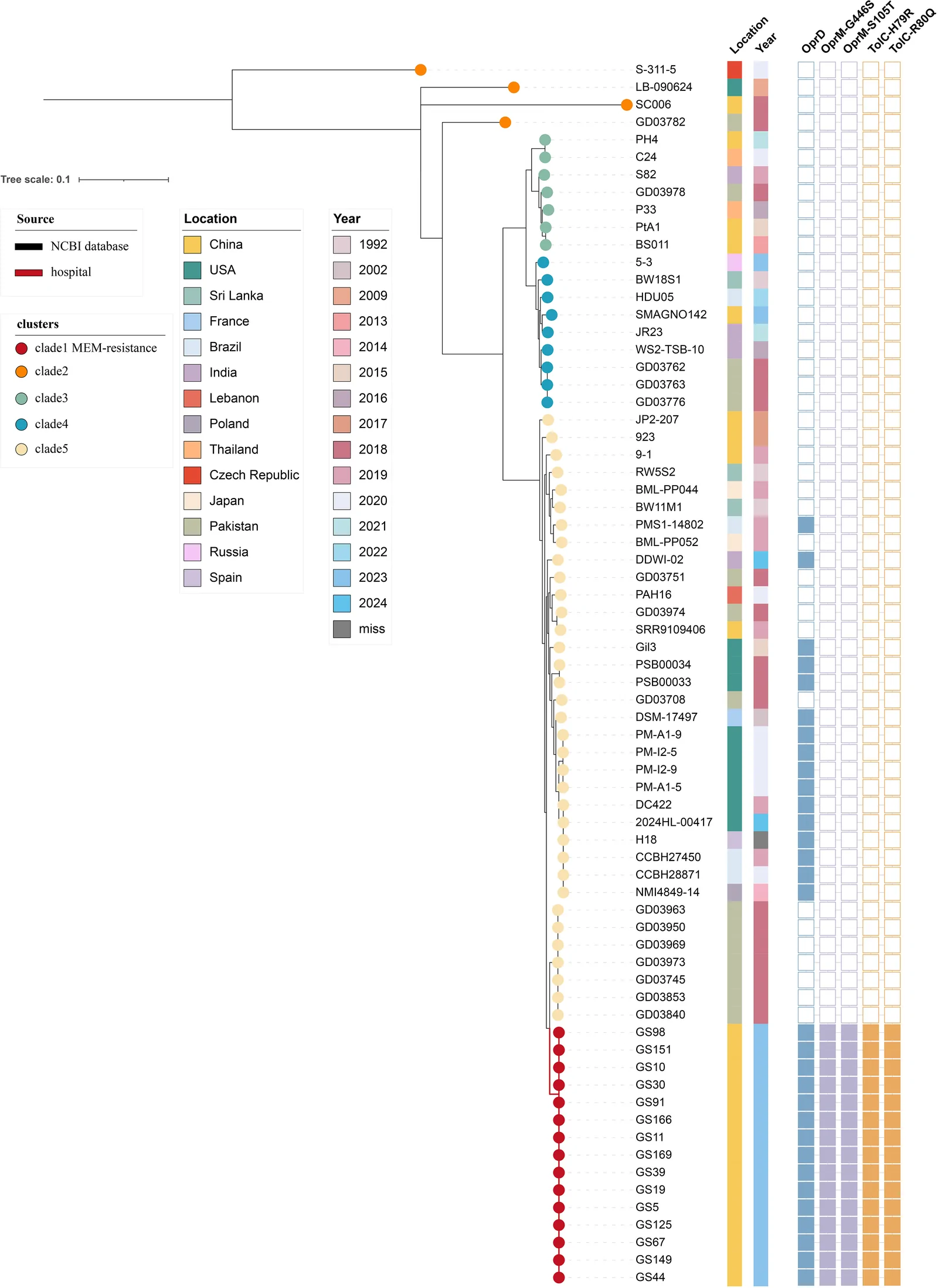
Phylogenetic tree based on SNP for *P. mosselii.* The inner circles represent different clusters. The outer small squares indicate the presence of outer membrane protein; filled squares denote the presence of the corresponding protein, while empty squares indicate its absence. Strains highlighted in bold red were collected from hospital in this study.

Genomic analysis was performed on the 15 selected *P. mosselii* isolates with reduced susceptibility to meropenem and confirmed susceptibility to imipenem by MIC testing. The *oprD* gene in all isolates remained intact, consistent with their phenotypic susceptibility to IPM as confirmed by MIC testing. Notably, two amino acid substitutions (S105T and G446S) were identified in the OprM protein. In addition, three-dimensional structures of the wild-type and mutant proteins were predicted and compared (Fig. 1). The S105T substitution in the cap domain involves the replacement of Ser with Thr. Although both are polar amino acids, the additional methyl group in threonine may alter the local hydrophobicity of the cap region. Furthermore, the G446S mutation occurs within the α-helical bundle. The transition from Gly to Ser introduces a hydroxyl-containing side chain, which potentially disrupts the inter-helical hydrogen-bonding network or causes steric hindrance. Meanwhile, its regulatory genes (*mexR*, *nalC*, and *nalD*) and the MexAB efflux pumps were not detected in any of the analyzed genomes. Again, nonsynonymous mutations were detected in several efflux pump-related genes. Specifically, two substitutions (H79R and R80Q) were found in the TolC protein, a V357A variation was identified in an RND family efflux protein, and a T31A substitution was observed in the TtgC protein (Table 2). These mutations are all located within components of outer membrane factors or efflux pump complexes, potentially altering local protein conformation or surface charge distribution and thereby influencing efflux activity. Comparative SNP analysis and sequence alignment of publicly available *P. mosselii* genomes in the NCBI database revealed that a small subset of strains harbored the same RND family substitution (T31A) as well as *oprD*. Furthermore, SNP-based phylogenetic analysis indicated that the 15 selected isolates recovered from hospitalized patients were highly homologous (SNPs ≤ 13), suggesting a clonal relationship. All strains included in the FastBAPS analysis, comprising the 15 selected isolates recovered from this study and 55 publicly available *P. mosselii* genomes retrieved from the NCBI database, were clustered into five clades (Fig. 2). However, the specific mechanisms by which these mutations contribute to MEM susceptibility require further functional validation.

**TABLE 2:** Amino acid substitutions in membrane-associated efflux and porin proteins among *P. mosselii* isolates^*a*^

	Amino-acid substitution(s)				
Strain	OprD (A0A0A7PUA7)	OprM (GCA_000621225.1)	TolC (A0A2D0AJB8)	RND (A0A246GR08)	TtgC (A0A2K4XH92)
GS10	WT	G446S, S105T	H79R, R80Q	V357A	T31A
GS11	WT	G446S, S105T	H79R, R80Q	V357A	T31A
GS125	WT	G446S, S105T	H79R, R80Q	V357A	T31A
GS149	WT	G446S, S105T	H79R, R80Q	V357A	T31A
GS151	WT	G446S, S105T	H79R, R80Q	V357A	T31A
GS166	WT	G446S, S105T	H79R, R80Q	V357A	T31A
GS169	WT	G446S, S105T	H79R, R80Q	V357A	T31A
GS19	WT	G446S, S105T	H79R, R80Q	V357A	T31A
GS30	WT	G446S, S105T	H79R, R80Q	V357A	T31A
GS39	WT	G446S, S105T	H79R, R80Q	V357A	T31A
GS44	WT	G446S, S105T	H79R, R80Q	V357A	T31A
GS5	WT	G446S, S105T	H79R, R80Q	V357A	T31A
GS67	WT	G446S, S105T	H79R, R80Q	V357A	T31A
GS91	WT	G446S, S105T	H79R, R80Q	V357A	T31A
GS98	WT	G446S, S105T	H79R, R80Q	V357A	T31A

^*a*^ WT, wild type; G, glycine; S, serine; T, threonine; H, histidine; R, arginine; Q, glutamine; V, valine; A, alanine. Accession numbers in parentheses indicate the corresponding reference protein sequences.

**Fig 3:**
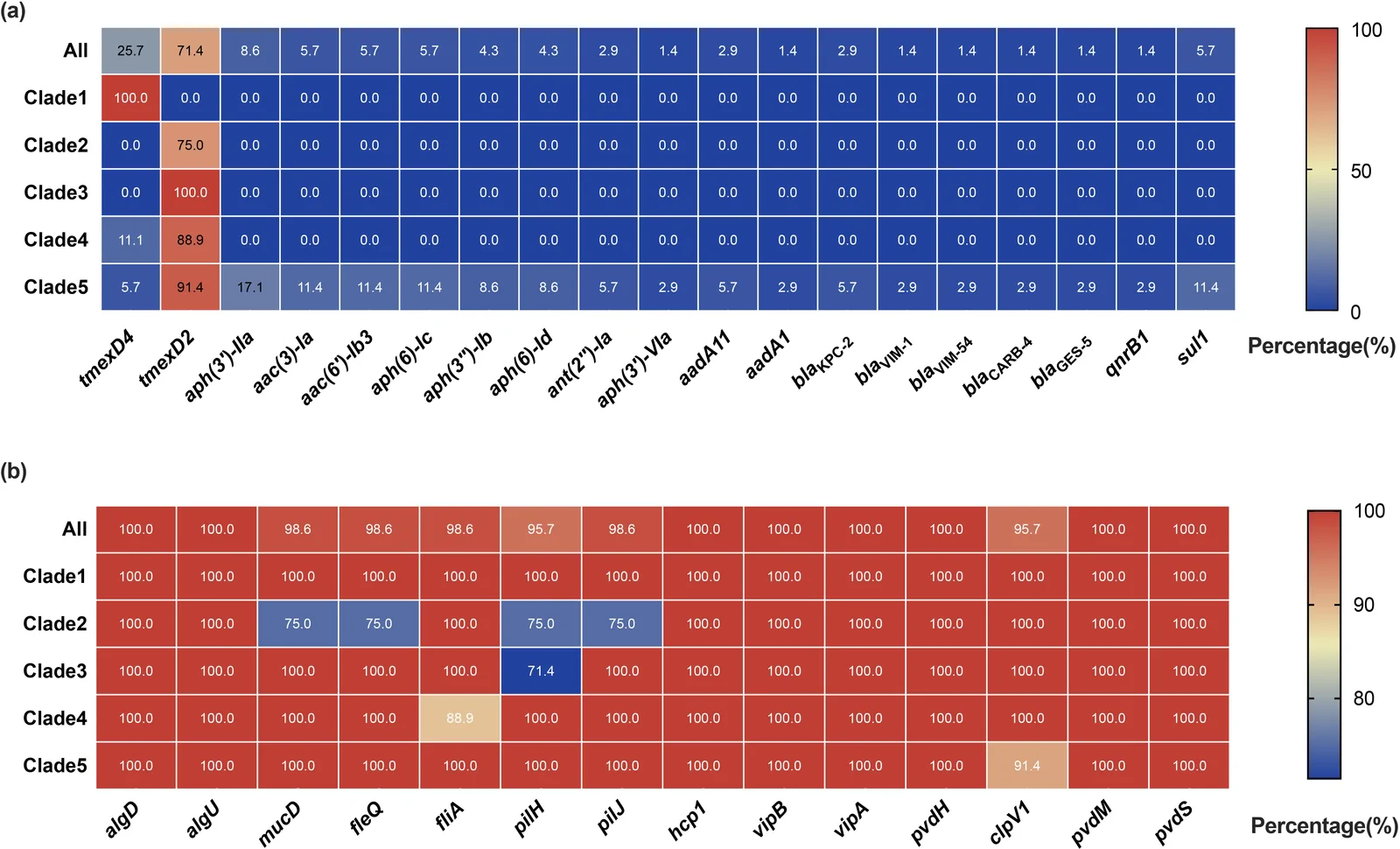
Percentage of clinical P. mosselii isolates carrying a particular antimicrobial resistance gene (a) or virulence-associated gene (b) across different phylogenetic clades.

The distribution of antimicrobial resistance genes varied among different phylogenetic clades (Fig. 3a). *tmexD4* was detected in all isolates of Clade 1, with a detection rate of 100%, whereas *tmexD2* was the most frequently detected resistance gene overall, being present in 71.4% of all isolates. Other acquired resistance genes were detected at relatively low frequencies, including aminoglycoside resistance genes such as *aph(3*′*)-II*a, *aac(3)-Ia*, *aac(6*′*)-Ib3*, *aph(6)-Ic*, *aph(3*″*)-Ib*, *aph(6)-Id*, *ant(2*″*)-Ia*, *aph(3*′*)-VIa*, *aadA11*, and *aadA1*, β-lactamase genes such as *bla*_KPC-2_, *bla*_VIM-1_, *bla*_VIM-54_, *bla*_CARB-4_, and *bla*_GES-5_, as well as *qnrB1* and *sul1.*

The distribution of virulence-associated genes among different phylogenetic clades is shown in the heat map (Fig. 3b). Virulence gene profiling revealed the consistent presence of key determinants related to alginate biosynthesis, including *algD*, *algU*, and *mucD*, across all 15 selected strains. Genes involved in flagellar regulation, such as *fleQ* and *fliA*, as well as type IV pili genes *pilH* and *pilJ*, were also detected. Components of the type VI secretion system, namely hcp1, *vipA*/*vipB*, and clpV1, were widely present, indicating a conserved T6SS architecture among the isolates. In addition, genes responsible for siderophore biosynthesis, including *pvdH*, *pvdM*, and *pvdS*, and those associated with Lipopolysaccharide (LPS) core assembly, such as *waaG* and *waaP*, were identified in the majority of strains.

## DISCUSSION

In this study, we observed an atypical phenotype in selected *P. mosselii* isolates, which displayed reduced susceptibility to MEM while remaining susceptible to IPM as confirmed by MIC testing. This phenomenon has never been reported previously although sporadic cases have been described in other opportunistic pathogens. For instance, studies in *P. aeruginosa* and *A. baumannii* have revealed dissociation between susceptibility and resistance to carbapenems (14, 15), suggesting that the underlying resistance mechanisms may be more complex than conventionally recognized.

Differences exist in the dependency of the two carbapenems on membrane permeability (16). Previous studies have demonstrated that the OprD porin plays a pivotal role in IPM uptake, whereas MEM exhibits relatively lower dependence on OprD (17). Thus, when the OprD channel remains intact, isolates may retain susceptibility to IPM despite resistance to MEM. Consistent with this, the *oprD* gene in the selected isolates remained intact, consistent with their susceptibility to IPM. Moreover, efflux pumps may contribute to the discrepancy between MEM and IPM. Certain members of the RND efflux pump family (e.g., MexAB–OprM) have been reported to extrude MEM more efficiently (18), thereby conferring enhanced resistance to MEM. In our study, genomic analysis identified two amino acid substitutions (S105T and G446S) in OprM. Previous studies have not reported cases where amino acid mutation sites in the OprM protein alone lead to MEM resistance. Most researches on the MexAB-OprM system-mediated resistance have focused on mutations in *mexB* or its regulatory genes, such as *mexR*, *nalC*, *nalD* (19). Specific amino acid substitutions in MexB, including K134N, G621S, and R620C, are located near the substrate-binding pocket of the efflux pump. These changes may alter substrate specificity or transport efficiency, thereby enhancing resistance to certain antibiotics (20). Similarly, mutations or abnormal expression of the regulator *nalC*, such as Gly71Glu and Ser209Arg, as well as alterations in *mexR*, have been shown to upregulate the MexAB–OprM system and contribute to MEM resistance (21, 22). However, neither the MexAB efflux pump genes nor the regulatory genes *mexR*, *nalC*, and *nalD* were detected in our study, suggesting that *P. mosselii* may rely on alternative efflux mechanisms distinct from those described in *P. aeruginosa*. Therefore, we analyzed other efflux-associated proteins, finding several nonsynonymous mutations in key components of alternative efflux systems. Specifically, two substitutions (H79R and R80Q) were identified in the TolC outer membrane protein, a V357A substitution was found in an RND family efflux protein, and a T31A substitution was observed in the TtgC transporter. These mutations are located within structural regions that may influence substrate recognition or channel conformation, thereby altering efflux efficiency or substrate specificity and potentially explaining the observed MEM-resistant phenotype. Studies have shown that *Klebsiella pneumoniae* strains exposed to MEM show increased expression of *acrA* and *tolC* (23), and inhibitors targeting this system can reduce resistance levels. In *Escherichia coli*, non-carbapenemase isolates with increased expression of AcrAB-TolC display elevated MEM MICs (24). Furthermore, we identified mutations in *tolC* in *P. mosselii*, which may contribute to the observed reduced susceptibility to MEM. To our knowledge, there are no reports so far that directly link TtgC to reduced susceptibility to MEM. The identification of nonsynonymous variation in TtgC among the selected *P. mosselii* isolates suggests a potential role for this transporter in the observed phenotype and represents a novel avenue for future investigation.

Virulence gene profiling in the selected isolates highlights the potential pathogenicity and environmental adaptability of *P. mosselii*. The presence of *algD*, *algU*, and *mucD* indicates a conserved alginate biosynthesis pathway that may facilitate biofilm formation and provide protection against host immune responses and antibiotic stress (25, 26). Similarly, *fleQ* and *fliA* function as key regulators of flagellar assembly and motility, enhancing the bacterium’s ability to colonize surfaces (27). The identification of type VI secretion system genes such as *hcp1*, *vipA*/*vipB*, and clpV1 suggests that *P. mosselii* may engage in interbacterial competition, while siderophore-associated genes including *pvdS*, *pvdH*, and *pvdM* reflect its capacity for efficient iron acquisition under nutrient-limited conditions (28). Taken together, these findings indicate that *P. mosselii* not only carries antimicrobial resistance determinants but also harbors a diverse array of virulence factors that may contribute to its persistence and clinical significance.

SNP analysis further showed that the 15 selected isolates with reduced susceptibility to meropenem shared high genetic similarity with each other (≤13 SNPs) but exhibited substantial genomic divergence from publicly available *P. mosselii* genomes in the NCBI database. This finding suggests that these clinical isolates form a distinct phylogenetic cluster, likely representing a locally evolved lineage rather than dissemination of a previously characterized strain. Notably, two isolates were recovered from newborns immediately after birth and one from a 2-month-old infant, indicating that nosocomial transmission within the hospital environment cannot be excluded. Such genetic distinctiveness, together with the occurrence of infections in neonates, underscores the potential for local dissemination and highlights the importance of genomic monitoring for early detection of emerging resistance lineages.

### Limitations

This study has several limitations. First, the screening strategy was based on reduced susceptibility to meropenem, which may have missed *P. mosselii* isolates that are susceptible to meropenem but exhibit decreased susceptibility to imipenem. However, the primary aim of this study was to characterize the antimicrobial resistance and virulence traits of selected isolates rather than to assess the prevalence of carbapenem resistance. Second, disk diffusion testing was used solely as an initial screening approach, as species-specific disk diffusion breakpoints are not available for *P. mosselii* in CLSI guidelines. Categorical resistance interpretations were, therefore, based exclusively on MIC testing using CLSI breakpoints for other non-Enterobacterales. Finally, the number of isolates included was limited due to strain availability although the consistent phenotypic and genomic characteristics observed provide valuable insight into potential resistance mechanisms and virulence features.

## MATERIALS AND METHODS

### Bacterial isolates

In 2023, 190 fecal samples were collected from hospitalized patients in a tertiary hospital in Gansu Province, China. These samples were initially screened on Mueller–Hinton agar plates supplemented with 0.2 μg/mL MEM to select for isolates with reduced susceptibility to meropenem. A total of 63 isolates were identified as *P. mosselii* using MALDI-TOF MS (Bruker Daltonik GmbH, Bremen, Germany).

### Antimicrobial susceptibility testing

Antimicrobial susceptibility testing for all strains was initially performed using the Kirby–Bauer disk diffusion method. Disk diffusion testing was used solely as a screening approach and not for categorical resistance interpretation, as CLSI disk diffusion breakpoints are not available for *P. mosselii.* Isolates showing reduced inhibition zone diameters to meropenem in disk diffusion testing were selected for further analysis. MICs of MEM and IPM were subsequently determined by the E-test, while the MICs of ticarcillin, piperacillin, ceftazidime, cefoperazone, cefepime, aztreonam, amikacin, tobramycin, ciprofloxacin, levofloxacin, doxycycline, minocycline, tigecycline, and trimethoprim were determined using the VITEK 2 Compact automated system with the VITEK 2 AST-N335 card (bioMérieux, France). MIC results were interpreted according to the Clinical and Laboratory Standards Institute (CLSI) M100-S33 breakpoints for Other non-Enterobacterales (29). *P. aeruginosa* ATCC 27853 was employed as the quality control strain.

### Whole-genome sequencing and genome analysis

For selected *P. mosselii* isolates exhibiting reduced susceptibility to MEM while remaining susceptible to IPM, the genomic DNA was subjected to WGS using the Illumina NovaSeq 6000 platform. Thereafter, the generated sequencing data were *de novo* assembled with SPAdes Version 3.13.1 (30). The identification of antimicrobial resistance genes (ARGs) within the assembly scaffolds was carried out employing ResFinder Version 4.1 (31) with default parameters. Reference sequences for the virulence genes were sourced from the VFDB databases (32). Heatmaps showing the distribution of ARGs, virulence genes, and selected genomic features were generated using GraphPad Prism (version 9.0.0 for Windows, GraphPad Software, Boston, Massachusetts, USA; www.graphpad.com). In addition, all publicly available *P. mosselii* genome sequences were downloaded from the NCBI database for comparative analysis (Table S1). For comparative genomic analysis, core-genome alignment, single-nucleotide polymorphism (SNP) calling, and the construction of SNP-based phylogenetic trees were performed using the Parsnp script (33). Then, an online tool, iTOL Version 3 (34), was used to systematically visualize and annotate all phylogenetic trees. And all the strains further clustered by FastBAPS v1.0.8 software (35) based on the core-genome SNP alignment. In this study, a cluster was defined as a distinct genetic subpopulation identified via the optimized symmetric prior model. This hierarchical Bayesian clustering approach partitions the population by maximizing the marginal likelihood of the sequence data. We specifically utilized the Level 1 clustering results, which represent the primary population partition, to define the major lineages of the *P. mosselii* strains. A gene catalog (Unigenes) was established by mapping and filtering genes. The DIAMOND software (version 0.9.9, available at https://github.com/bbuchfink/diamond/) (36) was employed to perform BLAST searches against the Unigenes extracted from the OMPdb database (accessible at http://www.ompdb.org) (37). Coding sequences that exhibited a high percentage of identity (>99%) and coverage (>90%) were subsequently identified. The three-dimensional structures of selected proteins were predicted using AlphaFold (38), and structural visualization and figure refinement were performed using PyMOL Molecular Graphics System (Version 3.0.0, Schrödinger, LLC).

## Data Availability

The whole-genome sequencing data set of 15 *P. mosselii* isolates was retrieved with BioProject accession number PRJNA1345955.

## SUPPLEMENTAL MATERIAL

Table S1 spectrum.03560-25-s0001.xlsx Reference strains for phylogenetic analysis. Detailed information of 55 reference strains used in phylogenetic analysis with their accessions.

ASM does not own the copyrights to Supplemental Material that may be linked to, or accessed through, an article. The authors have granted ASM a non-exclusive, world-wide license to publish the Supplemental Material files. Please contact the corresponding author directly for reuse.
